# An Indwelling Urethral Catheter Knotted Around a Double-J Ureteral Stent: An Unusual Complication after Kidney Transplantation

**DOI:** 10.1155/2011/672326

**Published:** 2012-01-18

**Authors:** E. G. Warmerdam, R. J. Toorop, A. C. Abrahams, P. Berger

**Affiliations:** ^1^Department of Vascular Surgery, University Medical Centre Utrecht, P.O. Box 85500, 3508 GA Utrecht, The Netherlands; ^2^Department of Nephrology and Hypertension, University Medical Centre Utrecht, P.O. Box 85500, 3508 GA Utrecht, The Netherlands

## Abstract

Urethral catheterization is a common procedure with a relatively low complication rate. Knotting of an indwelling urethral catheter is a very rare complication, and there are only a few case reports on knotted catheters, most of them concerning children. We report an especially rare case where a urethral catheter formed a knot around a double-J ureteral stent after a kidney transplantation. We will discuss the various risk factors for knotting of a catheter and the methods to untangle a knot.

## 1. Introduction

Urethral catheterization is a very common medical procedure and is associated with a relatively low complication rate. Complications mostly comprise urinary tract infections and urethral injury with bleeding and are usually mild in nature. A knot in an indwelling urethral catheter is a very uncommon complication of urethral catheterization with an estimated incidence of 2 per one million [1]. Very limited cases describing a knot in a urethral catheter have been reported, and, to the best of our knowledge, this report is the first in which a urethral catheter is knotted around a double-J ureteral stent.

## 2. Case Report

A 51-year-old woman was admitted to our transplant unit for a kidney transplantation. She had a medical history with Klippel-Feil syndrome (congenital cervical vertebral fusion) and Wegener's granulomatosis causing end-stage kidney disease for which hemodialysis was initiated 7 years ago. She had no residual renal function and was finally accepted for a kidney transplantation. She received a left kidney from a nonheart beating donor. The kidney had a normal anatomy that is, a single artery, vein, and ureter. General anaesthesia was given using propofol and sufentanil, and the patient was given a ch14 urethral catheter (Teleflex, Durham, NC, USA). According to standard procedure, the balloon was left high in the bladder to facilitate identification and exposure to the bladder during transplantation.

The kidney was transplanted in the right iliac fossa. The renal vein was anastomosed end-to-side to the external iliac vein. Thereafter, the renal artery was anastomosed end to side to the external iliac artery. Second warm ischemia time in recipient was 33 minutes. After reperfusion graft colour was homogenous and a normal Doppler, sound was audible on the renal cortex. A standard Lich-Gregoire anastomosis was created between the donor ureter and the recipient's bladder. A 6 F double-J stent (Urosoft, Murray Hill, NJ, USA) was left in the ureter and bladder, in order to protect the anastomosis.

**Figure 1:**
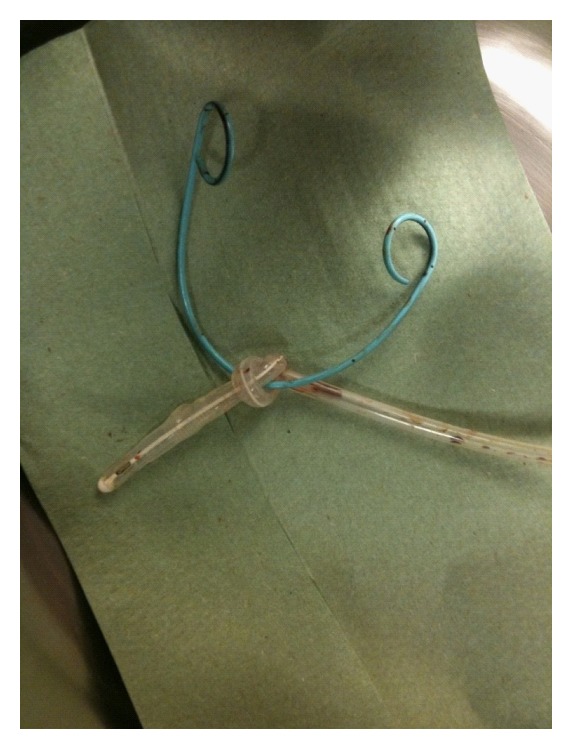
A Photograph taken after the removal of the urethral catheter, showing that the urethral catheter formed a knot around the double-J stent (blue).

Maintenance immunosuppression therapy consisted of steroids, tacrolimus, and mycophenolate mofetil. After the operation, the patient recovered well. According to our protocol, the urethral catheter was removed five days posttransplantation. This was done gently, and no abnormal resistance was noted. The patient did not experience pain. To our surprise, not only the urethral catheter, but also the double-J stent was removed. Apparently the catheter had formed a knot around the double-J stent (Figure 1). An ultrasound was made to make sure that no complications had occurred due to the removal of the knotted catheter and double-J stent. Standard postoperative duplex scanning showed a normal perfusion of the kidney. On day 10, posttransplantation plasma creatinine levels started to decrease. The patient was discharged from the hospital on day 14. Six weeks after transplantation plasma creatinine was 95 *μ*mol/L. No late complications occurred after the removal of double-J stent.

## 3. Discussion

The formation of knots in urethral catheters is a rare complication. Little is known about why some catheters develop a knot. One hypothesis is that excessive length of the catheter inside the bladder causes knotting. With decompression of the bladder, and the catheter tip can loop through a coil of the catheter [2, 3]. Another hypothesis is that the insertion of the catheter disturbs the surface tension of the fluid in the bladder, this results in a low pressure area around the tip thus creating a water current and facilitating knot formation [2]. When there is a significant length (at least 10 cm, according to Raveenthiran [2]) of catheter inside the bladder, the tip is able to swirl as a result of the water current and can then form a spontaneous open-loop knot. Other risk factors for knotting include small diameter catheters and overdistention of the bladder. Small diameter catheters tend to be more flexible, thereby possibly increasing the risk for knotting [3]. This might be the reason why most reports about catheter knots are from children [4, 5]. Overdistension (over 300 mL of urine) of the bladder results in a more violent water current, thereby, increasing the risk of knotting [2].

In our case, there are a couple of possibilities on how the urethral catheter was able to form a knot, thereby trapping the double-J stent. The most important way to avoid excessive catheter length inside the bladder is by gently pulling the catheter after insertion thereby positioning the balloon against the lower wall of the bladder. Since we routinely position the catheter high up in the bladder to facilitate exposure of the bladder during transplantation, it is well possible that we omitted to reposition the catheter balloon to the bladder wall immediately after the operation. The excess length of the urethral catheter could have facilitated knot formation. Trapping of the double-J stent may have been an unfortunate circumstance but may also have been caused by migration of the double-J stent down from the anastomosis, which is a known complication [6]. A larger part of the stent present in the bladder would have made it easier for the urethral catheter to form a knot around it.

The removal of a urethral catheter should always be done gently. Resistance during removal should raise suspicion of a possible complication, for example, a knot. The removal of the catheter by force has to be avoided and should be replaced with making a cystography. If a knot is suspected or detected, prior to the removal of the catheter, various techniques are described to remove the knotted catheter safely. These include manual removal after sustained traction under general anaesthesia; open cystotomy under general or local anaesthesia percutaneous cystotomy under general anaesthesia [1], and fluoroscopic manipulation of a guidewire to unknot the catheter in the bladder [7].

Our case illustrates that even low-risk procedures can have serious complications. Avoiding excessive length of catheter inside the bladder is essential to minimize the risk of a knot being formed.
